# Agonistic *β*2-Adrenergic Receptor Autoantibodies Characterize the Aqueous Humor of Patients With Primary and Secondary Open-Angle Glaucoma

**DOI:** 10.3389/fimmu.2021.550236

**Published:** 2021-05-05

**Authors:** Bettina Hohberger, Max Wörn, Robert Lämmer, Aparna Mahajan, Christian Mardin, Ursula Schötzer-Schrehardt, Rudolph Kunze, Martin Herrmann, Gerd Wallukat

**Affiliations:** ^1^ Department of Ophthalmology, Friedrich-Alexander-University of Erlangen-Nürnberg, Erlangen, Germany; ^2^ Department of Internal Medicine III, Institute of Clinical Immunology and Rheumatology, University of Erlangen-Nürnberg, Erlangen, Germany; ^3^ Science office, Berlin-Buch, Campus Max Delbrück Center for Molecular Medicine, Berlin, Germany; ^4^ Max Delbrück Center for Molecular Medicine, Berlin, Germany

**Keywords:** autoantibodies, autoimmunity, glaucoma, aqueous humor, *ß*2-adrenergic receptor

## Abstract

**Purpose:**

Agonistic *β*2-adrenergic receptor autoantibodies (*β*2-agAAbs) were recently observed in sera of patients with ocular hypertension (OHT), primary (POAG), and secondary open-angle glaucoma (SOAG), yet not in healthy controls (HCs). It was the aim of the present study to investigate the presence of *β*2-agAAb in aqueous humor (AH) samples of OAG patients and to correlate these with the corresponding *β*2-agAAb serum data.

**Material and Methods:**

Thirty-nine patients (21 male, 18 female) were recruited from the Department of Ophthalmology, University of Erlangen-Nürnberg: twenty-one POAG, 18 SOAG. Aqueous humor samples were collected during minimal invasive glaucoma surgery. Serum and AH samples were analyzed for *β*2-agAAb by a bioassay quantifying the beating rate of cultured cardiomyocyte (cut-off: 2 U).

**Results:**

Thirty-six of 39 (92.3%) and 34 of 39 (87.2%) of OAG patients showed a *β*2-agAAb in their sera and AH samples, respectively. All *β*2-agAAb AH-positive OAG patients were also seropositive. We also observed a *β*2-agAAb seropositivity in 95 and 89% of patients with POAG and SOAG, respectively. Beta2-agAAbs were seen in 86% (POAG) and 78% (SOAG) of AH samples. The *β*2-agAAb adrenergic activity was increased in the AH of patients with POAG (6.5 ± 1.5 U) when compared with those with SOAG (4.1 ± 1.1 U; p = 0.004). Serum *β*2-agAAb adrenergic activity did not differ between the cohorts [POAG (4.5 ± 1.5 U); SOAG (4.6 ± 2.1 U; p=0.458)]. No correlation of the beating rates were observed between serum and AH samples for group and subgroup analyses.

**Conclusion:**

The detection of *β*2-agAAb in systemic and local circulations supports the hypothesis of a direct functional impact of these agAAbs on ocular G-protein coupled receptors. The high prevalence of *β*2-agAAb in serum and AH samples of patients with POAG or SOAG suggests a common role of these AAbs in the etiopathogenesis of glaucoma, independent of open-angle glaucoma subtype.

## Introduction

Glaucoma is as neurodegenerative disease, one of the leading causes for blindness in the developing countries. Its pathogenesis is assumed to be multifactorial with an increased intraocular pressure (IOP) as main risk factor. All therapeutic options—either conservative, laser or surgical methods—aim to lower the increased IOP up to an individual defined target level. Yet, all patients show a disease progression despite an optimal level of IOP (1). Thus, it is general accepted that several other factors are involved in the complex multifactorial pathophysiology of glaucoma. In addition to vascular dysregulation (2), oxidative stress (3–5), and abnormalities of the lamina cribrosa (6) and the trabecular meshwork (7), the involvement of autoimmune mechanisms (8–10) are seen as risk factors.

In 2018 agonistic autoantibodies against the adrenergic *β*2 receptor (*β*2-agAAb) were initially identified in sera of patients with ocular hypertension (OHT; n = 9) and primary open-angle (POAG; n = 39) glaucoma, yet not in healthy controls (n = 17) (11). A recent study showed the presence of these agonistic *β*2-agAAb in sera of patients with secondary OAG (SOAG; n = 11) as well (12). The presence of *β*2-agAAb in patients with OHT and OAG suggests a fundamental involvement of these autoantibodies in the pathogenesis of increased ocular pressure and glaucoma, respectively.

Beta2-adrenergic receptors (*β*2-ARs) were shown to be present on non-pigmented epithelial cells of the ciliary body (13, 14) and endothelial cells of the trabecular meshwork (14, 15), both involved in the regulation of IOP. These identified *β*2-AR autoantibodies are directed against the second extracellular loop of the G-protein coupled *β*2-AR. Due to the agonistic activity of the *β*2-AAb, the *β*2-ARs are stimulated without showing a receptor desensitization, usually observed using pharmaceutical *β*2-AR agonists (*e.g.* Clenbuterol). Consecutively, the *β*2-AR is stimulated chronically (12). We hypothesize that this chronic stimulation of the *β*2-AR by *β*2-agAAb influences both the production of aqueous humor (AH) by ciliary epithelial cells and the outflow resistance in the trabecular meshwork (TM) resulting in an increased IOP.

The molecular pathway may be mediated by the Na-K-Cl co-transporter in both ocular tissues. In TM the endothelial cell volume is proposed to be regulated by Na-K-Cl co-transporter (16). *In vitro* data support this assumption as cell volumes changed after stimulation or inhibition of the Na-K-Cl co-transporter (16). After hyperactivation of Na-K-Cl co-transporter in TM cells osmotic changes might induce an increased cell volume, consequently decreased extracellular space and decreased outflow of AH. Data of Putney et al. support this hypothesis, showing an increased volume of TM cells in glaucoma patients (17). In the ciliary epithelium a non-selective *β*2-AR blocker (isoproterenol) reportedly stimulates the Na-K-Cl co-transporter (18) and, consequently, the chloride transport (19). Moreover, AH flow was increased after application of epinephrines (20). Thus, we assume that hyperactivation of Na-K-Cl co-transporter in cells of the ciliary body might induce an increased AH flow, as observed in eyes with open-angle glaucoma especially at nighttime (21). This hypothesis of an influence of *β*2-AAb on the increased IOP is supported by *in vivo* data showing a decrease in IOP and/or number of local antiglaucomatous eye drops after wash-out of *β*2-agAAb by unspecific immunapheresis (12). As all recent studies were based on *β*2-agAAb positivity, it would be of interest if these agonistic autoantibodies would also be present locally in aqueous humor (AH). A potential presence of *β*2-agAAb in AH would support our hypothesis of a direct impact of functional active *β*2-agAAb on ocular G-protein coupled receptors (GPCRs). In addition, a potential hint for the primary production of *β*2-agAAb might be offered *via* correlation of AH and sera data. Thus, it was the aim of the present study to investigate the presence of these *β*2-agAAbs in AH samples of patients with OAG in correlation with their corresponding serum data.

## Material and Methods

### Patients

Thirty-nine subjects were recruited from the Department of Ophthalmology, University of Erlangen-Nürnberg (21 male, 18 female): twenty-one POAG and 18 SOAG. Demographic data are summarized in **Table 1**. All patients with OAG were under local antiglaucomatous therapy and required a minimal invasive glaucoma surgery (MIGS), as antiglaucomatous therapy was not tolerated and/or IOP was not regulated under maximal local antiglaucomatous therapy. Each patient underwent a complete ophthalmological examination including measurement of best corrected visual acuity and IOP, measured by Goldmann applanation tonometry (Haag Streit, Koeniz, Switzerland). Slit-lamp biomicroscopy of the anterior segment [configuration of the anterior chamber angle, presence of melanindispersion and/or pseudo-exfoliation (PEX)] was performed. Funduscopy was done with grading of the optic disc according to the classification after Jonas (22). Visual field was measured by standard white-on-white full-field perimetry (Octopus 500, G1 protocol, mean defect, MD, Interzeag, Schlieren, Switzerland). Additionally, global retinal nerve fiber layer (gRNFL) was measured by Spectral Domain Spectralis II OCT (Heidelberg Engineering, Heidelberg, Germany). Serum and aqueous humor samples were acquired during the same stay in the hospital. The study was approved by the local ethic committee (53_14B) and performed according to the tenets of the Declaration of Helsinki. Informed consent was received from each patient, respectively.

**Table 1:** Demographic data: age, gender, best corrected visual acuity (BCVA), intraocular pressure (IOP, mmHg), number of local antiglaucomatous eye drops, mean defect (MD) and global retinal nerve fiber layer (gRFNL, µm) for open-angle glaucoma (OAG), primary OAG (POAG) and secondary OAG patients (SOAG); mean ± standard deviation, p-value.

	Total OAG	POAG	SOAG	p-value
**Age**	69.72 ± 8.9	69.62 ± 7.5	69.83 ± 10.5	>0.05
**Gender (female/male)**	18/21	10/11	8/10	>0.05
**BCVA**	0.59 ± 0.2	0.58 ± 0.2	0.61 ± 0.2	>0.05
**IOP**	22.22 ± 7.5	22.30 ± 8.8	22.12 ± 5.8	>0.05
**Number of local medication**	2.59 ± 0.9	2.85 ± 1.1	2.29 ± 0.5	0.042
**MD**	11.38 ± 6.8	12.51 ± 6.9	9.96 ± 6.7	>0.05
**Global RNFL**	65.06 ± 20.4	60.85 ± 19.1	70.31 ± 21.3	>0.05

### Aqueous Humor Sampling

AH samples were obtained during MIGS. Before cutting the corneal tunnel, aqueous humor samples were received *via* ab-externo limbal paracentesis by a 27-gauge needle on a tuberculin syringe. The samples were stored at −80°C.

### POAG Eyes

Diagnosis of POAG was based on an open anterior chamber angle, repeated (≥two times) measurements of increased IOP > 21 mmHg (measured with Goldmann applanation tonometry), glaucomatous alterations of the optic disc [according to the classification after Jonas (22)], and visual field loss (confirmed at least once).

### SOAG Eyes

Diagnosis of SOAG was based on an open anterior chamber angle, repeated (≥two times) measurements of increased IOP > 21 mmHg (measured with Goldmann applanation tonometry), glaucomatous alterations of the optic disc [according to the classification after Jonas (22)], and visual field loss (confirmed at least once). In addition, SOAG eyes showed melanindispersion in the anterior chamber (melanin dispersion glaucoma) and/or PEX material on structures of the anterior chamber (PEX glaucoma).

### Cardiomyocyte Bioassay

The cardiomyocyte bioassay was performed as described previously (12). Cell cultures of cardiac myocytes were established by digestion of tissue samples (heart ventricle of 1–2 day-old Sprague–Dawley rats) with a 0.25% solution of crude porcine trypsin (Serva, Germany). The cells were dispersed and suspended in SM20-I medium (Biochrom, Germany), containing glutamine (Serva, Germany), streptomycin (HEFA Pharma; Germany), penicillin (Heyl, Germany), hydrocortisone (Merck, Germany), 10% heat-inactivated neonatal calf serum (Gibco, Germany), and fluorodeoxyuridine (Serva, Germany). The cells were seeded at a density of 160,000 cells/cm^2^. After 24 h the culture medium was replaced. Before stimulation the cardiomyocytes were cultured for 3–4 days (37°C). 2 h before the experiment, the medium was renewed. The sera and aqueous humor samples were dialyzed in order to remove small bioactive components, which could potentially be present in the sample. The dialysation did not attach the IgG fraction. Afterwards the samples were applied directly to the cardiomyocyte bioassay. This method was chosen on the basis of a previous experiment. In a trial (n = 32) the adrenergic activity, measured by the bioassay, was observed to be the same for IgG isolated (done by direct ammonium sulfate precipitations described previously (12) and dialyzed sera (p = 0.179, t-test): adrenergic activity in the bioassay after applying IgG, isolated by ammonium sulfate precipitations: 5.40 ± 0.28 U (after incubation with ICI118.51 0.1 µM: 0.24 ± 0.06 U); adrenergic activity in the bioassay after applying the dialyzed sera: 4.92 ± 0.21 (after incubation with ICI118.551 0.1 µM: 0.09 ± 0.05 U).

The beating rates of 7–10 spontaneously beating cardiomyocytes were counted for 15 s at a heated stage inverted microscope (37°C). An increase of 8 beats/s is considered 1 unit of adrenergic activity (U). Samples below two units of adrenergic activity were considered as normal.

### Statistical Analysis

Statistical analysis was done by SPSS version 21.0 (IBM, New York, USA). Data were presented as mean ± standard deviation. Data were tested for normality previous to subsequent statistical analysis. Non-parametric test was used for subgroup analysis of serum adrenergic activity of *β*2-agAAb. T-test was used for subgroup analysis of AH adrenergic activity of *β*2-agAAb. Additionally, Qui-quadrat test and Pearson (for analysis of normal distributed data) and Spearman (for analysis of non-normal distributed data) correlation analyses were done for group and subgroup analyses. Demographic data were analyzed by t-test, non-parametric test and Fisher exact test.

## Results

### *β*2-agAAb-Seropositivity

**Figure 1:**
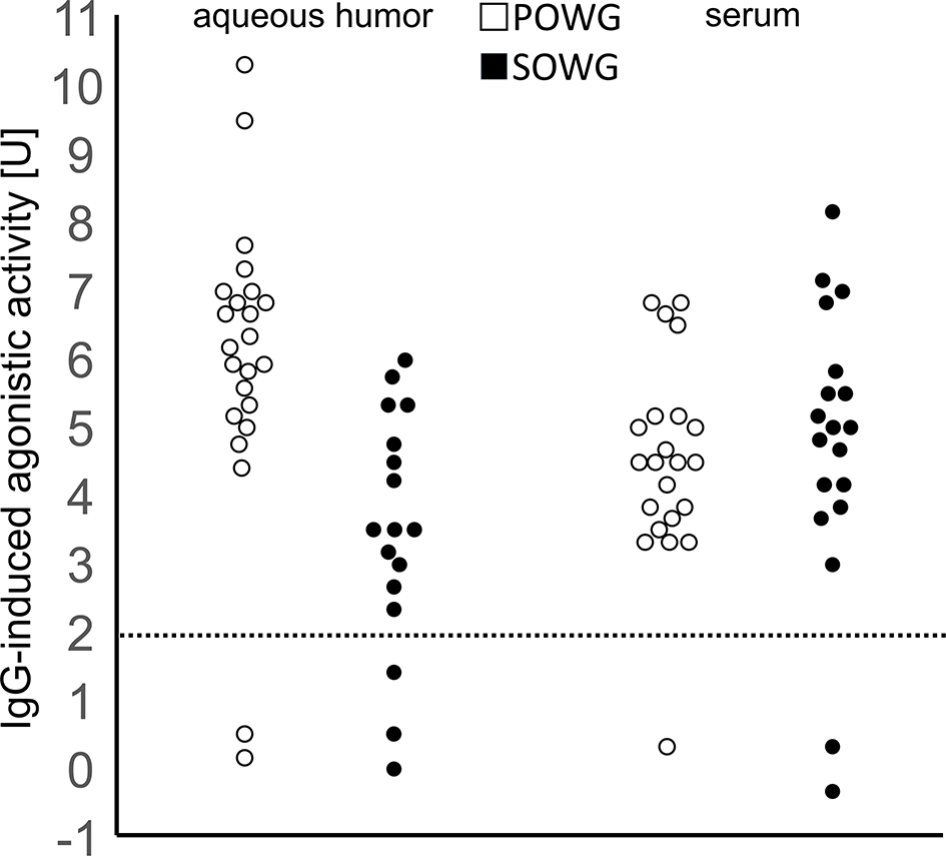
Agonistic activity of *β*2-agonistic autoantibodies.Stimulation of cardiomyocytes by sera or aqueous humor samples from patients with OAG. Open dots: POAG, filled dots: SOAG; normal range <2 units adrenergic activity. Statistics: all POAG against all SOAG p = 0.035; all AH against sera p = 0.96; POAG AH against sera p = 0.09; SOAG AH against sera p = 0.047; AH POAG against SOAG p = 0.003; sera POAG against SOAG p = 0.727.

Beta2-agAAb-seropositivity was observed in 36 of 39 OAG patients (92.3%). Mean beating rate of *β*2-agAAb seropositive OAG patients was 4.60 ± 1.8 U. Subgroup analysis yielded a *β*2-agAAb in 95% of sera of POAG and 89% of sera of patients with SOAG (**Figure 1**). Beta2-agAAb seropositive POAG eyes showed a mean beating rate of 4.5 ± 1.5 U. A mean beating rate of 4.6 ± 2.1 U was measured in *β*2-agAAb seropositive patients with SOAG. No statistically significant differences were observed by subgroup analysis (p = 0.458).

### *β*2-agAAb AH-Positivity

Beta 2-agAAbs were found in 34 of 39 AH samples of patients with OAG (87.2%). All patients (34/34) with *β*2-agAAb in their AH were also seropositive (100%). Subgroup analysis yielded a *β*2-agAAb AH-positivity in 86 and 78% of patients with POAG and SOAG, respectively (**Figure 1**). No significant differences were observed for *β*2-agAAb-AH-positivity between patients with POAG and SOAG (p = 0.506).

A mean beating rate of 4.8 ± 2.4 U was measured in AH of *β*2-agAAb AH-positive eyes from patients with OAG. Subgroup analysis showed 5.9 ± 2.3 U and 3.6 ± 1.7 U in AH-positive eyes from patients with POAG and SOAG, respectively. Accordingly, *β*2-agAAb AH adrenergic activity was significantly increased in patients with POAG as compared to those with SOAG (p = 0.001).

### Association of *β*2-agAAb Adrenergic Activity in Serum and AH

**Figure 2:**
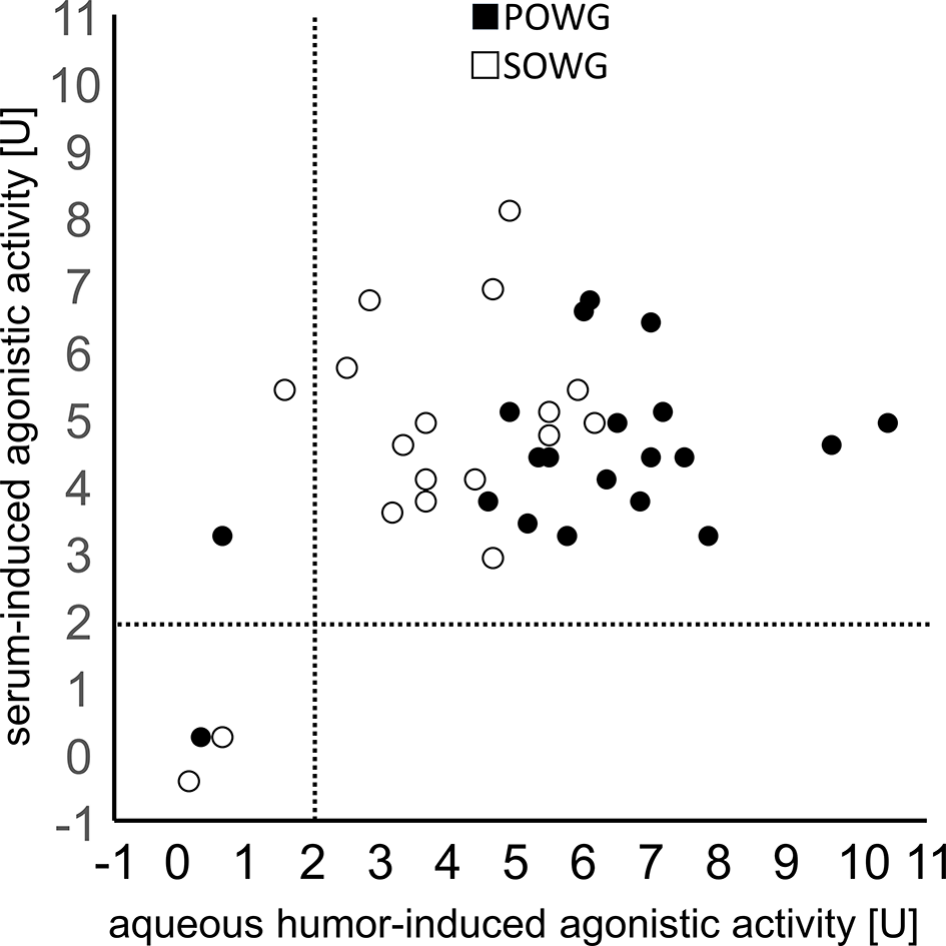
Correlation of the agonistic activities of *β*2-agonistic autoantibodies in sera and aqueous humor. AH samples and corresponding sera of patients with POAG (filled dots) or SOAG (open dots) are displayed. Note, the activity values are mutually independent (p > 0.05, Spearman correlation).

No statistically significant differences were observed for mean beating rates of *β*2-agAAb seropositive and *β*2-agAAb AH-positive OAG eyes (p = 0.202). Importantly, *β*2-agAAb adrenergic activity in AH and the corresponding sera were independent and did not correlate significantly (Spearman correlation, **Figure 2** and **Table 2**).

**Table 2:** Correlation analysis of agonistic activities of *β*2-agAAb in aqueous humor (AH) with their corresponding *β*2-agAAb in sera, intraocular pressure (IOP), mean defect (MD), and global retinal nerve fiber layer (gRFNL) for open-angle glaucoma (OAG), primary OAG (POAG), and secondary OAG patients (SOAG, exact p-values are shown): *β*2-agAAbs were observed to be independent.

			β2-agAAbin sera	β2-agAAbin AH	IOP	MD	gRNFL
**β2-agAAb**	**OAG**	**in sera**	–	0.119	0.624	0.164	0.234
		**in AH**	0.119	–	0.339	0.930	0.180
	**POAG**	**in sera**	–	0.154	0.158	0.256	0.137
		**in AH**	0.154	–	0.338	0.575	0.363
	**SOAG**	**in sera**	–	0.189	0.518	0.511	0.636
		**in AH**	0.189	–	0.833	0.979	0.960

Subgroup analysis for *β*2-agAAb AH- and seropositive patients with OAG yielded a significantly increased beating rate in AH samples compared to serum samples in POAG (p = 0.001), yet only slightly in patients with SOAG (p = 0.043). Further analyses did not show any significant correlation of *β*2-agAAb beating rate of AH and serum samples (**Table 2**).

### Association *β*2-agAAb Adrenergic Activity With Clinical Glaucoma Parameters

Individual serum adrenergic activity of *β*2-agAAb did not show any correlation with IOP, mean visual field defect, and global RNFL thickness in group and subgroup analyses (**Table 2**), respectively. No significant correlations were to be observed for AH adrenergic activity of *β*2-agAAb and IOP, mean visual field defect, and global RNFL for group and subgroup analysis (**Table 2**).

## Discussion

Autoimmune mechanisms are supposed to be involved in the pathogenesis of glaucoma (23–25). Several studies support the hypothesis of a contribution of immune mechanisms to the complex interactions of glaucoma risk factors (11, 26, 27). Based on previous observations of specific *β*2-agAAb in sera of OAG patients and their potential involvement in IOP elevation (12), it was of interest, to analyze whether these AAbs are also present locally in the AH. The data of the present study provide the first evidence for the presence of *β*2-agAAb in AH of patients with OAG. Their presence showed independent production of the AH antibodies since their adrenergic activity did not correlate with the corresponding sera data. Yet, glaucoma subtype seemed to be an influencing factor.

In the present study, *β*2-agAAbs were measured by an established cardiomyocyte bioassay, which represents a direct measure of AAb activity by alteration of the beating rate of cultured cardiomyocytes. In addition to the mere detection of the AAb, this technique enables testing their agonistic functionality. The *β*2-agAAbs activate the *β*2-receptors and thus enhanced the contractility of cultured cardiomyocytes. The time response curve of theses *β*2-agAAbs continued for even 5 h without desensitization, hereby leading to a substantial hyperactivation of the *β*2-receptor (not shown) (12).

Up to now, the *β*2-agAAbs were observed in sera of patients with OAG (12). The presence of these specific agonistic AAb in AH might support the above mentioned hypothesis of a regulatory capability of IOP by the local *β*2-agAAb. All patients with OAG, showing presence of *β*2-agAAb in AH, were also *β*2-agAAb seropositive. This observation is in accordance with previous observations of significantly increased Immunoglobulin G levels in AH of an experimental autoimmune glaucoma model (27). Two candidate mechanisms underly this observation: (I) the *β*2-agAAbs are either selectively imported into the eye alternatively; (II) they are produced locally. Due to a significantly increased agonistic activity in AH samples of POAG compared to those from SOAG, the data might suggest that the *β*2-agAAbs are produced locally in ocular tissues in POAG, yet, in SOAG the *β*2-agAAbs in the AH are derived from serum. As *β*2-agAAb adrenergic activity of AH and the corresponding serum turned out to be independent, we tend to speculate that the AH-borne *β*2-agAAbs are produced locally in ocular tissues.

Several autoimmune diseases show an involvement of extrafollicular immune responses (*e.g.* systemic lupus erythematousus, SLE) (28). Patients with SLE have an increased risk to develop glaucoma (adjusted IRR 2.2) (29). Different mechanisms were discussed in this context: the application of glucocorticoids (30), neuroendocrine-immune pathogenic alterations (31), and ectopic germinal centers as niches for antibody-secreting plasma cells (ASA) (28).

Canonical germinal centers are formed by B-cells after antigen contact and T cell help. These activated B-cells migrate to secondary lymphoid organs and pass the T-cell–B-cell border. In the center of the follicle the B-cells initiate the formation of a nascent germinal center (32). When these mature, they can morphologically be divided into a souring light zone (LZ) and inner dark zone (DZ). After somatic hypermutation (SHM) of the immunoglobulin (Ig) in the DZ, the B-cells that have developed high-affinity B cell receptors capture their specific antigen on the surfaces of follicular dendritic cells, process it, and present it CD4^+^ T helper cells (33). The affinity-maturated cells then home to the bone marrow where they mature to plasma cells that produce huge amounts of antibodies. It is discussed that the Ig itself, the antigen-antibody complex or secondary molecular cascades might be pathogenetic factors. Interestingly, in chronically inflamed tissues non-canonical, ectopic germinal center-like structures and tissue-borne plasma cells can develop. The data of the present study might suggest that the *β*2-agAAbs were produced locally in ocular tissues as the result of a local ectopic germinal center formation. Further studies might follow this hypothesis in order to visualize non-canonical germinal center-like structures in the ocular tissues of patients with glaucoma. *β*2-agAAbs are one of several autoantibody species (*e.g.* anti-HSP^70^, anti-vimentin) that have been detected in AH of patients with glaucoma (27, 34, 35). The levels of the former AAb could either be increased or decreased in patients.

Specific AAb patterns that reportedly depend on the glaucoma subtype are under investigation (36). As *β*2-agAAb adrenergic activity was observed in AH of POAG and SOAG and as these did not correlate with the routine glaucoma follow-up parameters, these data might indicate that *β*2-agAAb are involved in very early stages of glaucoma pathogenesis by shared pathophysiologic mechanisms. This hypothesis is supported by the clinical finding that not all patients with melanindispersion or PEX material show an increased IOP or glaucoma characteristics during follow-ups. Thus, additional risk factors are assumed to be involved in the pathogenesis of SOAG.

The study is not without limitations. Sample size is small; thus, subsequent studies might follow this issue in a larger patient cohort. In addition, it would be of interest, if these autoimmune phenomena are also present in sera and/or AH of glaucoma eyes of different ethnicities. As we are working on setting up an assay to measure the *β*2-agAAb concentration, correlation of the *β*2-agAAb level and routine glaucoma follow-up parameters would be of interest, when available.

If subsequent studies could observe the presence of non-canonical germinal center-like structures in ocular tissue of patients with glaucoma, this local production of *β*2-agAAb could be the basis for the development of a novel antiglaucomatous eye drop.

## Conclusion

The present study confirmed the high prevalence of *β*2-agAAb in sera of patients with OAG and, for the first time reported their presence in aqueous humor. A local production in the eye might be supposed that the adrenergic activity in aqueous humor did not correlate to those in their corresponding sera. The data of the present study might suggest the contribution of a common autoimmune response to the etiopathogenesis of glaucoma.

## Data Availability Statement

The raw data supporting the conclusions of this article will be made available by the authors, without undue reservation.

## Ethics Statement

The studies involving human participants were reviewed and approved by the ethics committee of Erlangen. The patients/participants provided their written informed consent to participate in this study.

## Author Contributions

BH, RK, MH, and GW had the idea. BH, MH, and GW planned the study. GW performed the laboratory work. RL, CM, and MW did the clinical trial. MW, BH, and MH performed data acquisition and statistical analysis. BH was responsible for the draft of the manuscript. AM US-S, and MH interpreted results and edited the manuscript. All authors contributed to the article and approved the submitted version.

## Funding

The Erlangen Glaucoma Registry was funded by the German Research Society (DFG) from 1991 to 2009, NCT00494923. No specific funding was achieved for the present study. Data are available upon request. No potentially identifiable human images or data is presented in this study.

## Conflict of Interest

RK, patent EP1832600A1; MH, patent EP1832600A1; and GW, Berlin Cures, patent EP1832600A1.

The remaining authors declare that the research was conducted in the absence of any commercial or financial relationships that could be construed as a potential conflict of interest.
